# Slow Receptor Dissociation Kinetics Differentiate Macitentan from Other Endothelin Receptor Antagonists in Pulmonary Arterial Smooth Muscle Cells

**DOI:** 10.1371/journal.pone.0047662

**Published:** 2012-10-15

**Authors:** John Gatfield, Celia Mueller Grandjean, Thomas Sasse, Martine Clozel, Oliver Nayler

**Affiliations:** Actelion Pharmaceuticals Ltd., Allschwil, Switzerland; Tohoku University, Japan

## Abstract

Two endothelin receptor antagonists (ERAs), bosentan and ambrisentan, are currently approved for the treatment of pulmonary arterial hypertension (PAH), a devastating disease involving an activated endothelin system and aberrant contraction and proliferation of pulmonary arterial smooth muscle cells (PASMC). The novel ERA macitentan has recently concluded testing in a Phase III morbidity/mortality clinical trial in PAH patients. Since the association and dissociation rates of G protein-coupled receptor antagonists can influence their pharmacological activity *in vivo*, we used human PASMC to characterize inhibitory potency and receptor inhibition kinetics of macitentan, ambrisentan and bosentan using calcium release and inositol-1-phosphate (IP_1_) assays. In calcium release assays macitentan, ambrisentan and bosentan were highly potent ERAs with K_b_ values of 0.14 nM, 0.12 nM and 1.1 nM, respectively. Macitentan, but not ambrisentan and bosentan, displayed slow apparent receptor association kinetics as evidenced by increased antagonistic potency upon prolongation of antagonist pre-incubation times. In compound washout experiments, macitentan displayed a significantly lower receptor dissociation rate and longer receptor occupancy half-life (ROt_1/2_) compared to bosentan and ambrisentan (ROt_1/2_∶17 minutes versus 70 seconds and 40 seconds, respectively). Because of its lower dissociation rate macitentan behaved as an insurmountable antagonist in calcium release and IP_1_ assays, and unlike bosentan and ambrisentan it blocked endothelin receptor activation across a wide range of endothelin-1 (ET-1) concentrations. However, prolongation of the ET-1 stimulation time beyond ROt_1/2_ rendered macitentan a surmountable antagonist, revealing its competitive binding mode. Bosentan and ambrisentan behaved as surmountable antagonists irrespective of the assay duration and they lacked inhibitory activity at high ET-1 concentrations. Thus, macitentan is a competitive ERA with significantly slower receptor dissociation kinetics than the currently approved ERAs. Slow dissociation caused insurmountable antagonism in functional PASMC-based assays and this could contribute to an enhanced pharmacological activity of macitentan in ET-1-dependent pathologies.

## Introduction

Pulmonary arterial hypertension (PAH) is a rare and severe disease that is characterized by increased pressure in the pulmonary circulation caused by progressive pulmonary artery remodeling and constriction of the pulmonary vasculature [1]. The initiating cause of this devastating disease is largely unknown; however, pulmonary endothelial dysfunction and smooth muscle cell abnormality are significant contributors [2].

Treatments that aim at restoring the balance of endothelium-derived vasoactive substances are effective in reducing pulmonary vascular resistance and increasing exercise capacity [3], [4], [5]. Such treatments comprise prostacyclin analogues, phosphodiesterase type 5 inhibitors and endothelin receptor antagonists (ERAs). Endothelin (ET) concentrations are elevated in lung tissue of PAH patients [6] and the central pathogenic role of ET in PAH has been demonstrated in several clinical trials evaluating different ERAs [7].

Endothelins are vasoactive peptides of which endothelin-1 (ET-1) is the most abundant in lung tissue [8]. ET-1 is the most potent and long lasting vasoconstrictor known in man, and it acts as mitogen, angiogenic factor, mediator of fibrosis and inflammation. All of these processes are aberrantly activated in pulmonary vessels in PAH [9], [10], [11], [12], [13]. ET-1 responses are mediated via activation of two homologous G protein-coupled receptor subtypes, endothelin A receptor (ET_A_) and endothelin B receptor (ET_B_) [14], [15]. Both receptor subtypes activate Gαq protein-mediated pathways leading to phospholipase Cβ activation and increased intracellular calcium concentrations [16].

Endothelial cells are the main source of ET-1 and they secrete this peptide via two routes. The constitutive pathway is thought to contribute to basal vascular tone whereas the non-constitutive pathway releases ET-1 in response to a variety of external stimuli from specialized storage vesicles [17], [18]. Consistent with its site of action, ET-1 secretion by endothelial cells is polarized towards the basolateral space, i.e. into the deeper tissues of the vessel wall [19]. The secretion of endothelial ET-1 is triggered by stimuli such as hypoxia, growth factors, cytokines, steroids, flow/shear stress and vessel injury [20], [21], [22], [23], [24], [25]. In the lung, changes in local ET -1 concentration are then sensed by neighboring pulmonary arterial smooth muscle cells (PASMC) and fibroblasts expressing ET receptors. In PASMC, ET receptors are coupled to the Gαq pathway and activate phospholipase Cβ and inositol-trisphosphate (IP_3_) and diacylglycerol production. These early second messengers initiate a biphasic calcium response resulting in sustained elevation of intracellular calcium levels. Elevated calcium levels promote of cytoskeletal contraction and cell proliferation [7], [16] and thereby mediate persistent vasoconstriction and vascular remodeling, both central pathological processes in PAH [26], [27], [28], [29].

In the present study we used second messenger assays in primary human PASMC to characterize ET receptor association and dissociation kinetics of macitentan, a novel ERA [30], in comparison with bosentan and ambrisentan, the two ERAs approved for the treatment of PAH. Macitentan had a slow apparent receptor association rate and a 15-fold extended receptor occupancy half-life (ROt_1/2_) compared to bosentan and ambrisentan. Consequently, macitentan, but neither bosentan nor ambrisentan, displayed insurmountable antagonism in cellular assays with short incubation times and blocked ET receptor activation irrespective of the added ET-1 concentration. Since blockade of ET receptor signaling in PASMC is a key aspect of successful therapeutic intervention in PAH, the unique kinetic properties of macitentan may allow a more effective treatment of the ET-1-mediated pathological hemodynamic and structural effects in PAH and other diseases.

## Results

### Contribution of Endothelin Receptor Subtypes to Signaling in PASMC

**Figure 1:**
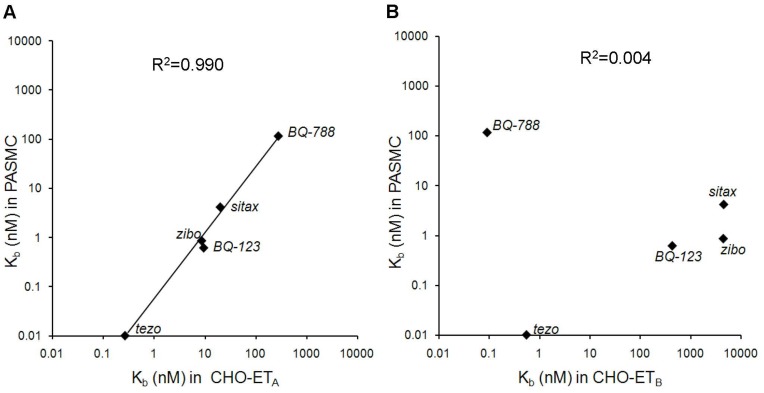
Contribution of endothelin receptor subtypes to calcium signaling in human primary PASMC. PASMC or CHO-ET_A_ or CHO-ET_B_ cells were pre-incubated with reference ERA dilution series for 120 min and then stimulated in the FLIPR with ET-1 (EC_50_–EC_70_). IC_50_ values were calculated from fluorescence peak responses and transformed into K_b_ values as described in Methods. Correlation scatter plot of K_b_ values obtained in PASMC versus CHO-ET_A_ cells (A) or versus CHO-ET_B_ cells (B). The correlation line and R^2^ value was generated by linear regression of the double-logarithmic data. Geometric means of n = 3–8 determinations are shown.

Cultured human primary PASMC, a central therapeutic target cell in PAH, expressed high levels of ET_A_ mRNA and considerably less ET_B_ mRNA (unpublished data and [28]). A set of five reference ERAs with different receptor subtype selectivity profiles was used to determine if the different receptor subtype mRNA expression levels were also reflected in the relative ET_A_ and ET_B_ receptor contribution to ET-1-induced calcium signaling. The antagonist K_b_ values obtained in PASMC were compared with K_b_ values generated in CHO cells expressing either the recombinant ET_A_ receptor or the recombinant ET_B_ receptor. To this end, antagonist dilution series were pre-incubated for 120 min with the respective cell types, which were then stimulated with ET-1 (EC_50_–EC_70_). Fluorescence traces were recorded for 3 min in the fluorescence imaging plate reader (FLIPR), and peak fluorescence values were used to calculate half maximum inhibitory concentrations of the antagonists (IC_50_). These were then transformed into functional inhibition constants (K_b_) using the Cheng-Prusoff equation [31] (see Methods). As shown in table 1, in recombinant cells, the dual ET_A_/ET_B_ receptor antagonist tezosentan displayed mixed ET_A_ and ET_B_ antagonism while the ET_A_ receptor antagonists BQ-123, sitaxentan and zibotentan selectively blocked ET_A_ receptors and the ET_B_ receptor antagonist BQ-788 selectively blocked ET_B_ receptors. In PASMC, the compounds covered a range of antagonistic potencies ranging from K_b_ = 10 pM for tezosentan to K_b_ = 117 nM for BQ-788. To assess the receptor subtype contribution to ET-1-induced signaling in human PASMC, the K_b_ values obtained in PASMC were compared with K_b_ values obtained in the CHO-ET_A_ system (Fig. 1A) or in the CHO-ET_B_ system (Fig. 1B). A significant correlation (R^2^ = 0.990) was obtained with CHO-ET_A_ data, while no correlation (R^2^ = 0.004) was seen with the CHO-ET_B_ data, suggesting that the ET_A_ receptor was the main mediator of ET-1-induced calcium release in these PASMC. A general shift to lower antagonistic potency was observed in the CHO-ET_A_ system, possibly due to receptor over-expression and a higher receptor reserve in the recombinant system. Tab. 1 summarizes these K_b_ data.

**Table 1:** Mean K_b_ values for reference ERAs determined by calcium release assays in CHO-ET_A_, CHO-ET_B_ and human PASMC.

	K_b_ (nM)*		
	CHO-ET_A_	CHO-ET_B_	PASMC
BQ-123	9.4	425	0.62
BQ-788	277	0.090	117
sitaxentan	20	4468	4.2
tezosentan	0.26	0.50	0.010
zibotentan	8.5	4389	0.87

* geometric means from n = 3–8 determinations calculated from IC_50_ values via Cheng-Prusoff equation (see Methods).

### Antagonistic Potency and Apparent Association Kinetics of Different ERAs

**Figure 2:**
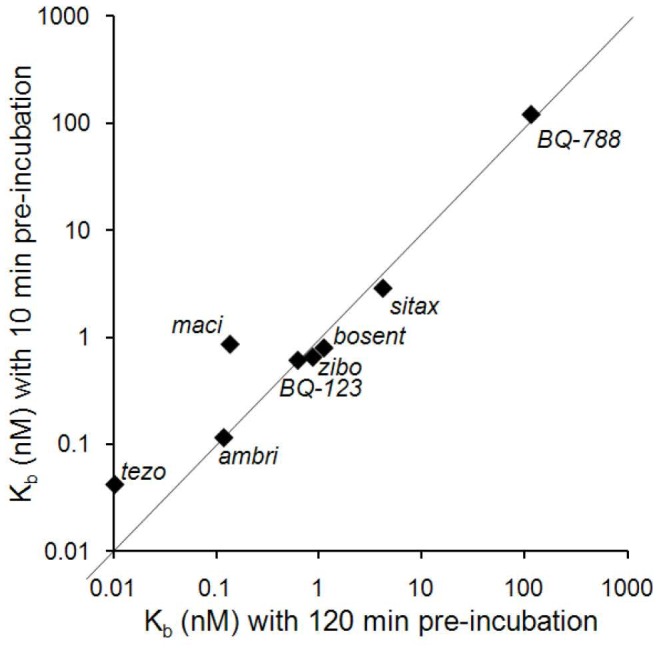
Antagonistic potency and apparent association kinetics of different ERAs using calcium release assays in PASMC. Cells were pre-incubated with antagonist dilution series for 10 min or 120 min and then stimulated in the FLIPR with ET-1 (EC_50_–EC_70_). IC_50_ values were calculated from fluorescence peak responses and transformed into K_b_ values as described in Methods. Correlation scatter plot of the K_b_ values generated for 10 min versus 120 min antagonist pre-incubation times (geometric means of n = 6 determinations).

Macitentan was compared with different known ERAs, including ambrisentan and bosentan, with respect to potency and apparent association kinetics in PASMC. The cells were pre-incubated with ERA dilution series for either 10 min or 120 min (steady state condition) and the resulting calcium signals were measured after stimulation with ET-1. The calculated K_b_ values for each compound and each incubation condition (Table 2 and Fig. 2) illustrate that all tested ERAs, with the exception of macitentan and to a lower extent of tezosentan, reached steady state after 10 min, as evidenced by the lack of potency shift when the pre-incubation time was extended from 10 min to 120 min. Macitentan however, displayed a 6.3-fold increase in potency after 120 min, compared to 10 min pre-incubation, indicating slow apparent association kinetics. In terms of potency ambrisentan and macitentan were similar at steady state after 120 min of pre-incubation (K_b_maci_ = 0.12 nM, K_b_ambri_ = 0.14 nM) while bosentan was approximately 10-fold less potent (K_b_bosent_ = 1.1 nM).

**Table 2:** Mean K_b_ values for different ERAs determined by calcium release assays in human PASMC with 10 min or 120 min antagonist pre-incubation time.

	K_b_ (nM)10 min*	K_b_ (nM)120 min*	K_b_ratio
ambrisentan	0.11	0.12	1.0
bosentan	0.79	1.1	0.7
BQ-123	0.61	0.62	1.0
BQ-788	121	117	1.0
macitentan	0.86	0.14	6.3
sitaxentan	2.9	4.2	0.7
tezosentan	0.042	0.010	4.1
zibotentan	0.65	0.87	0.7

* geometric means of n = 6 determinations calculated from IC_50_ values via Cheng-Prusoff equation (see Methods).

### Dissociation Kinetics and Receptor Occupancy Half-life of Macitentan, Ambrisentan and Bosentan

**Figure 3:**
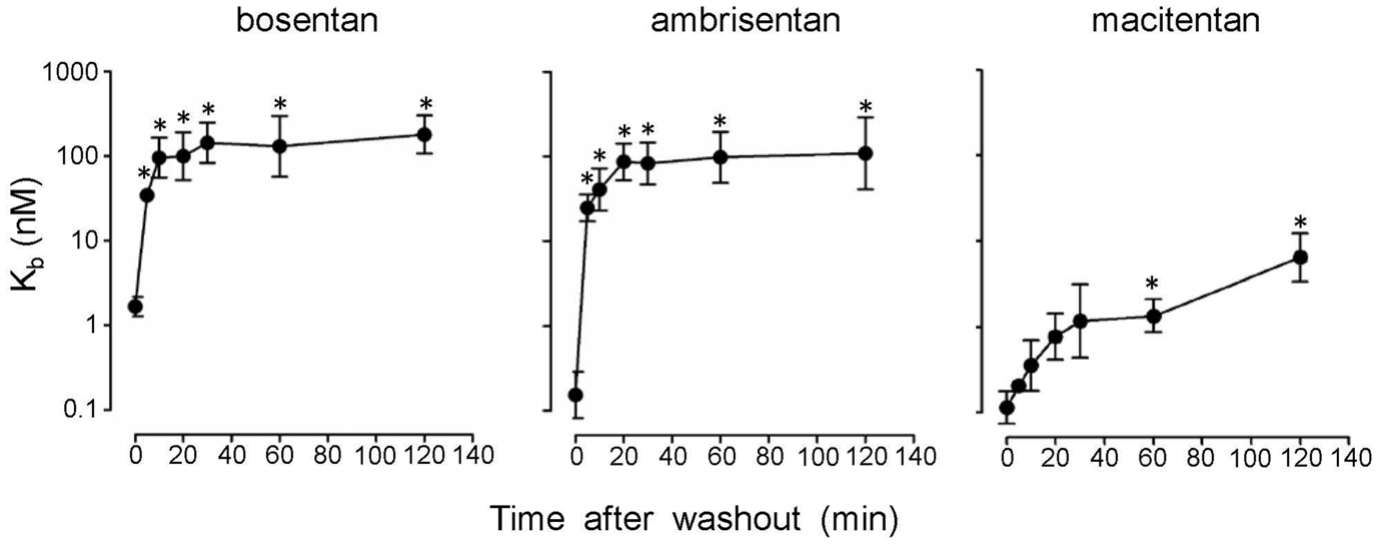
Dissociation kinetics of macitentan, ambrisentan and bosentan determined by calcium release assays in PASMC. Cells were pre-incubated for 120 min with antagonist dilution series and then either directly stimulated with ET-1 (EC_50_–EC_70_) or subjected to a compound washout procedure followed by stimulation with ET-1 at the indicated time points after washout. The peak calcium responses were used to calculate IC_50_ values which were transformed into K_b_ values as described in Methods. Shown are the time-dependent changes in K_b_ after washout. Geometric means are displayed +/− SEM. K_b_ values after washout significantly different (p<0.05) from the K_b_ at 0 min are indicated with an asterisk. Test: one-way ANOVA, Dunnett’s post test.

Slow apparent association kinetics can be caused by an increased receptor occupancy half-life (ROt_1/2_) and slow dissociation. Therefore, we determined the ROt_1/2_ of macitentan, bosentan and ambrisentan in PASMC using functional readouts after antagonist washout. PASMC were incubated with increasing concentrations of macitentan, ambrisentan or bosentan for 120 min and the cells were then either directly stimulated with ET-1 or subjected to a washout procedure to eliminate free compound. Following the washout procedure the cells were stimulated with an EC_50_–EC_70_ of ET-1 after different recovery times and calcium release was measured (Fig. 3). The IC_50_ values at the different time points were calculated and converted via the Cheng-Prusoff equation (K_b_ = IC_50_/(1+ [ET−1_stim_]/EC_50_ET−1_) to K_b_ values [31] using the EC_50_ of ET-1 determined on the same assay plate as well as the stimulating ET-1 concentration. At low stimulating ET-1 concentrations this calculation gives a good estimate of the K_b_ value even for insurmountable antagonists [32]. Five minutes after the compound was washed out, ambrisentan- and bosentan-treated PASMC had already regained their responsiveness to ET-1 stimulation, as evidenced by statistically significant shifts in K_b_ by factors of ∼170 (ambrisentan) and ∼20 (bosentan). In contrast, macitentan-treated PASMC remained ET-1 unresponsive and a statistically significant shift in K_b_ was first seen 60 min after washout. These data demonstrate a marked difference in the ROt_1/2_ of macitentan compared to ambrisentan, and bosentan. Based on the shift in K_b_ at 5 min, a ROt_1/2_ of ∼40 seconds (ambrisentan) and ∼70 seconds (bosentan) was calculated, assuming an exponential drop in receptor blockade over time. The 13-fold loss in potency of macitentan over 60 min translated to a ROt_1/2_ of ∼17 min. Taken together, macitentan showed a ∼15-fold increased ROt_1/2_ compared to ambrisentan and bosentan.

### Consequences for Mode of ET Receptor Antagonism

**Figure 4:**
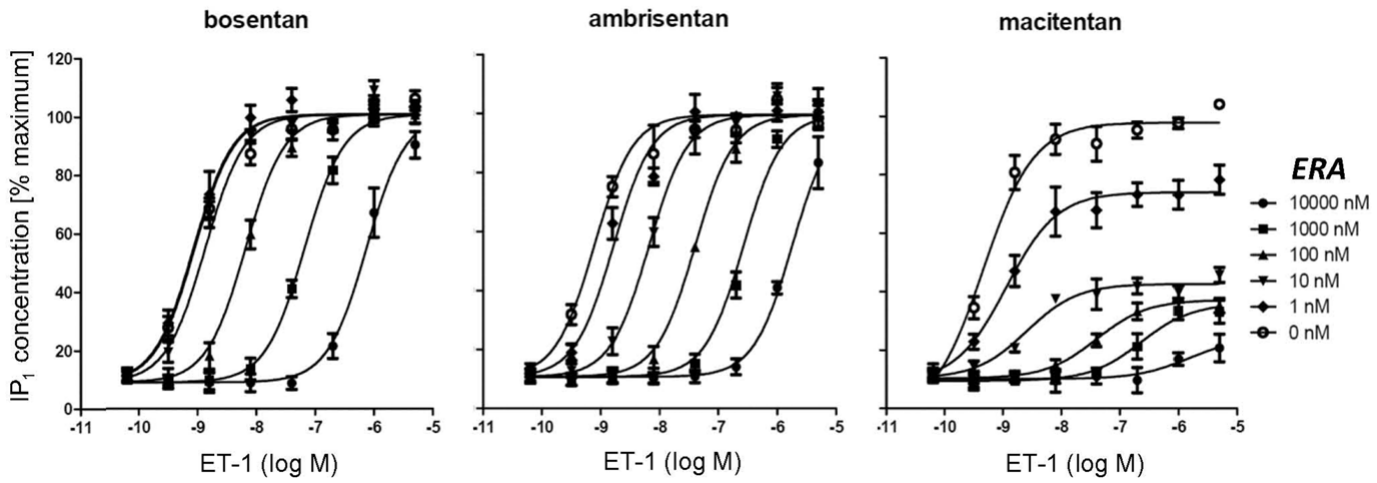
Effect of bosentan, ambrisentan and macitentan on ET-1-induced IP_1_ accumulation. Human PASMCs were pre-incubated with dilution series of antagonists for 120 min followed by the addition of a dilution series of ET-1. After 20 min of stimulation, cells were lysed and the IP_1_ content was determined. Raw data from 4 independent experiments were normalized, and average values +/− SEM are shown.

Compounds with increased ROt_1/2_ such as macitentan can behave as insurmountable antagonists when using agonist stimulation times that are shorter than the ROt_1/2_ of the antagonist-receptor complex [32]. IP_1_ accumulation assays are well suited to vary agonist stimulation times due to a long-lasting signal development and they are therefore often used to investigate the mode of antagonism of Gαq-coupled G protein coupled receptors. To assess whether macitentan, ambrisentan and bosentan differ in their mode of antagonism, concentration-response curves (CRCs) of ET-1 were generated in the presence of increasing concentrations of the three ERAs (Schild analysis), and IP_1_ accumulation was determined after 20 min of ET-1 stimulation. All three antagonists concentration-dependently blocked ET-1-induced IP_1_ accumulation in PASMC (Fig. 4). However, while ambrisentan and bosentan showed a surmountable mode of antagonism causing equidistant rightward shifts in the ET-1 CRCs, macitentan displayed an insurmountable mode of antagonism with a depression of maximum response in addition to rightward shifts in the ET-1 CRCs. Thus, the increased ROt_1/2_ of macitentan was associated with an insurmountable antagonism.

The IP_1_ accumulation assay data was also used to deduce K_b_ values for the different antagonists via the Cheng-Prusoff equation. Again, for K_b_ calculations we used the IC_50_ values that were generated at the lowest ET-1 concentration that delivered a robust signal strength (1.6 nM ET-1). Ambrisentan (K_b_: 1.1 nM, σ_g_ = 1.6, n = 4) and macitentan (K_b_: 0.74 nM, σ_g_ = 1.4, n = 4) showed similar potency and both were approximately 10-fold more potent than bosentan (K_b_: 12 nM, σ_g_ = 2.2, n = 4) with σg being the geometric standard deviation.These results were therefore in line with the data obtained by calcium flux assays in human PASMCs.

**Figure 5:**
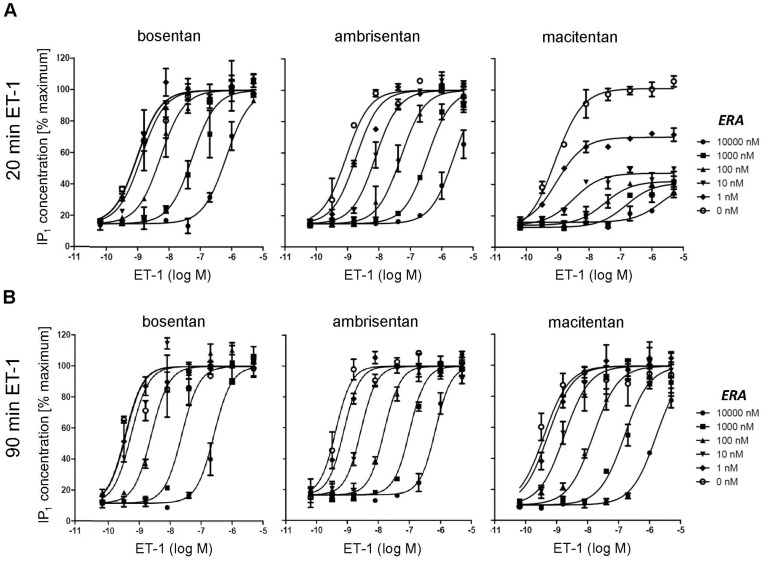
Time dependence of macitentan insurmountability determined in IP_1_ accumulation assays. Human PASMCs were pre-incubated with dilution series of antagonists for 120 min followed by the addition of a dilution series of ET-1. After 20 min (A) or 90 min (B) of stimulation, cells were lysed and the IP_1_ content was determined. Shown are the raw data +/− SD of duplicates of one representative experiment of a total of n = 3.

To differentiate slow-offset orthosteric antagonism from irreversible antagonism or allosteric antagonism, we next performed assays with longer stimulation times. Slow-offset antagonists behave as surmountable competitive antagonists in such assays if the stimulation time is significantly longer than the ROt_1/2_ of the antagonist-receptor complex. In contrast, allosteric antagonists or irreversible blockers would not change their mode of inhibition upon prolonged incubation. The IP_1_ accumulation assay was therefore performed with 20 minutes and with 90 minutes of ET-1 stimulation time, the latter being well beyond the estimated ROt_1/2_ of ∼ 17 min for macitentan and allowing for better antagonist-agonist equilibration. Macitentan displayed insurmountable antagonism with 20 minutes incubation time (Fig. 5A) and surmountable antagonism (Fig. 5B) when the stimulation time was prolonged to 90 minutes. In contrast, ambrisentan and bosentan displayed surmountable antagonism irrespective of the incubation time (Fig. 5A,B). Therefore, under equilibrium conditions macitentan revealed its competitive antagonistic mode of action as evidenced by equidistant rightward shifts of the ET-1 CRCs, full surmountability of the antagonism and a lack of saturability of the antagonistic effect that would be seen for negative allosteric modulators.

As surmountable antagonism was seen for all compounds for the 90-minute incubation time Schild K_b_ values could be calculated. As seen in previous experiments, macitentan (Schild K_b_: 1.4, σ_g_ = 2.1, n = 3) and ambrisentan (Schild K_b_ = 1.6 nM, σ_g_ = 2.3, n = 3) displayed similar potency and both were approximately 10-fold more potent than bosentan (Schild K_b_:16 nM, σ_g_ = 1.8, n = 3).

In summary, macitentan was a slow-offset orthosteric antagonist causing an insurmountable antagonism if the assay incubation time was shorter than the calculated ROt_1/2_ but a surmountable antagonism was observed if the assay time was longer than the calculated ROt_1/2_. In contrast, both ambrisentan and bosentan showed surmountable antagonism irrespective of the ET-1 stimulation times.

**Figure 6:**
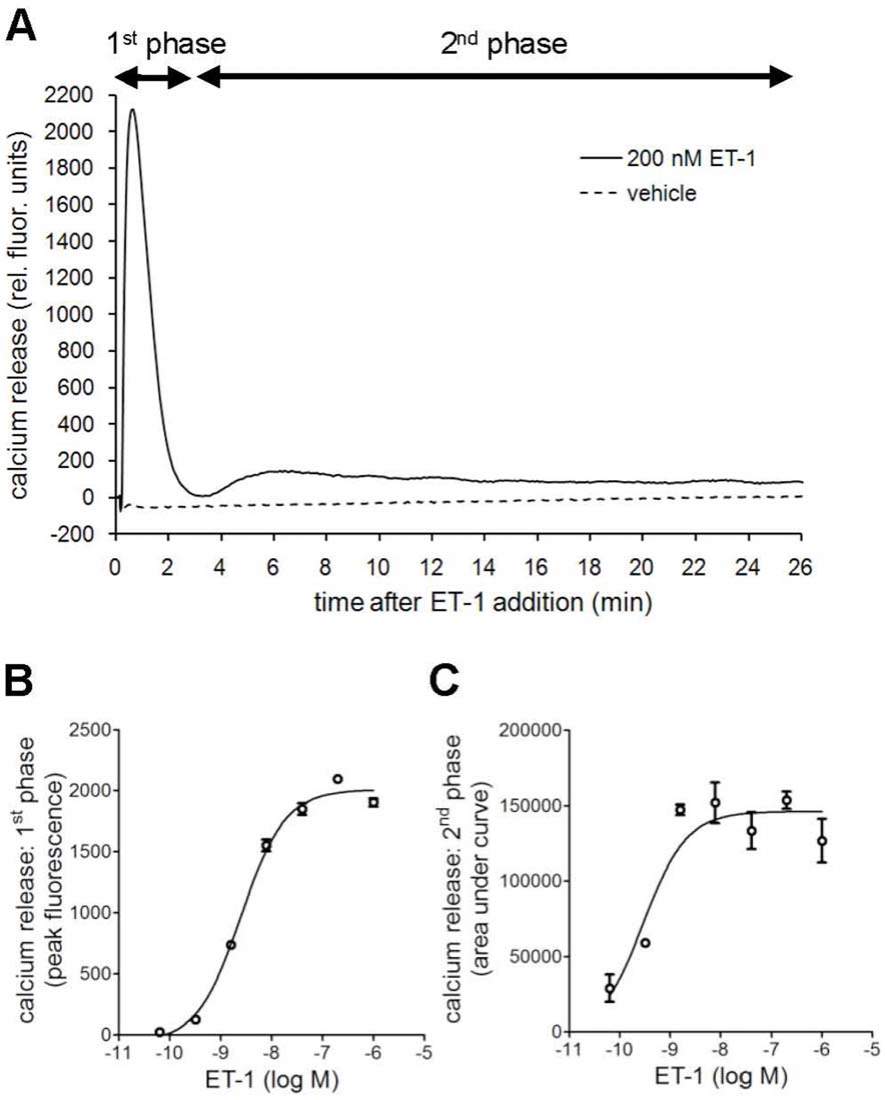
Characterization of the biphasic intracellular calcium response to ET-1 in PASMC. Cells were stimulated with a dilution series of ET-1 and the biphasic calcium response was recorded for 25 minutes. Shown are the raw FLIPR fluorescence traces for 200 nM ET-1 and vehicle treatment covering the first transient response phase and the second sustained response phase (A), the concentration-response-curves of ET-1 obtained for the first phase using peak fluorescence values +/− SD of duplicates (B) and the concentration-response-curves of ET-1 obtained for the second phase using area under the curve values between 3 min and 23 min after stimulation +/− SD of duplicates (C).

The calcium release induced in PASMC by ET-1 is characterized by a biphasic profile consisting of a rapid transient increase and a second sustained elevation [33], [34], [35], the latter being associated with sustained contraction and cell proliferation in pathology [29], [36]. We used these two response phases to further evaluate the three ERAs ambrisentan, bosentan and macitentan. In PASMC, ET-1 stimulation caused a robust, transient increase in cytosolic calcium, which peaked 30 seconds after ET-1 addition and lasted for about 3 min. It was followed by a second, prolonged but less pronounced increase in cytosolic calcium, which lasted for more than 25 min (Fig. 6A). Both phases showed a concentration-dependent, saturable behavior (Fig. 6B and C).

Macitentan, ambrisentan and bosentan were investigated in Schild experiments with 120-min pre-incubation times and the effects on the two phases of calcium increase were analyzed independently. The first response phase was quantified by using the fluorescence peak height within the first 3 minutes, and the second phase was quantified by calculating the area under the curve between 3 minutes and 23 minutes of observation.

**Figure 7:**
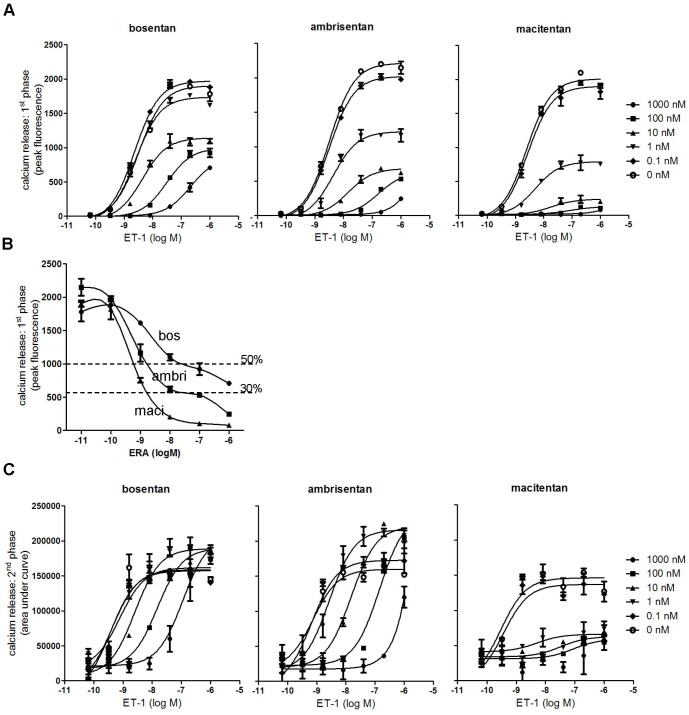
Effect of bosentan, ambrisentan and macitentan on both phases of the ET-1-induced calcium response. Human PASMCs were pre-incubated with dilution series of antagonists for 120 min, stimulated with a dilution series of ET-1 and the biphasic calcium response was recorded. Shown is the effect of antagonists on the ET-1 concentration-response-curves of the first response phase (A, using peak fluorescence values +/− SD of duplicates) and the second sustained response phase (C, using area under the curve values +/− SD of duplicates). Furthermore, the concentration-response-curves of the three antagonists for antagonizing peak responses induced by 1 µM ET-1 are shown +/− SD of duplicates (B) with the approximate localization of intermediate plateaus of antagonist efficacy. Shown are the results of one representative experiment out of n = 3 experiments.

Due to the fast signal development of the first calcium response (30 seconds to peak), all three compounds displayed a certain degree of insurmountable antagonism as expected, although macitentan was the most pronounced (Fig.7A). The extent of insurmountability was further illustrated by displaying the ERA inhibition curves at a fixed ET-1 concentration (1 µM). For ambrisentan and bosentan these curves were biphasic (Fig.7B) and reached an intermediate plateau of antagonistic efficacy with the residual unblocked signal being descriptive of the proportion of receptor that is subject to surmountable antagonism [37]. In fact, bosentan showed a surmountable mode of antagonism for ∼50% of the ET-1-induced signal and ambrisentan showed this surmountable mode for ∼30% of the ET-1-induced signal. Macitentan showed no surmountable behavior. Therefore, based on these observations it can be estimated that within the first 30 seconds, bosentan had dissociated from ∼50% of the receptors and ambrisentan from ∼30% of receptors (assuming a 1∶1 correlation between signal and receptor occupancy and lack of receptor reserve). These considerations yield a very short ROt_1/2_ of <1 minute for ambrisentan and bosentan.

Fig. 7C shows the antagonistic effects of the three compounds on the second sustained phase of calcium elevation. Macitentan displayed an insurmountable mode of antagonism while the other two compounds showed surmountable antagonism. Using the Cheng-Prusoff equation, the K_b_ values were calculated for the first (K_b1,_ using IC_50_ values at 8 nM ET-1) and second phase (K_b2_ using IC_50_ at 1.6 nM ET-1). Once again, macitentan (K_b1_∶0.17 nM and K_b2_ 0.093 nM) and ambrisentan (K_b1_∶0.27 nM and K_b2∶_0.14) were almost equipotent and ∼10-fold more potent than bosentan (K_b1_∶2.3 nM and K_b2∶_0.79). It is interesting to note that ambrisentan and bosentan treatment did not only lack inhibitory capacity on sustained calcium release at high ET-1 concentrations, but their presence reproducibly increased the maximal efficacy of ET-1 in this readout.

## Discussion

### Relevance of Drug-target Binding Kinetics

The pharmacological activity of a drug depends on target affinity and on pharmacokinetic variables such as free fraction and plasma half-life and on physicochemical properties that influence the speed and degree of compound distribution into the target tissue. All of these factors are now well established and are part of any medicinal chemistry compound optimization program. However, there is increasing evidence that the kinetic behavior of the drug–target complex also influences the clinical activity of a compound [38]. Prolonged target engagement is common among effective inhibitors and in fact, of the new molecular entities approved by the FDA between 2001 and 2004, only 18% of the orthosteric inhibitors or antagonists displayed a mechanism of inhibition following purely mass action competition, whereas the majority of inhibitors (70%) were capable of sustained target blockade [39]. Thus, sustained target blockade by slow dissociation is an effective strategy to avoid or at least delay the competition between an increase in physiological ligand concentration and the drug at the target binding site. In case of strongly fluctuating ligand concentrations at the target, which can be caused by local pulsatile secretion combined with rapid degradation, sustained target blockade can lead to enhanced maximal efficacy of an inhibitor.

Many autocrine and paracrine GPCR ligands display high temporarily restricted local concentrations, and sustained receptor blockade/insurmountable antagonism seems ideally suited to enhance maximal antagonistic efficacy in such systems. Well known examples of GPCR antagonists dealing with such fluctuating para/autocrine ligands are histamine H1 antagonists (e.g. desloratidine, ROt_1/2_∼6 h, [40]) for the blockade of allergic responses induced by mast-cell degranulation and P_2_Y_12_ antagonists (e.g. clopidogrel, irreversible binding, [41]) for the blockade of platelet aggregation induced by platelet-derived ADP. Another class of well-studied GPCR antagonists targeting an at least partially autocrine/paracrine system are the angiotensin II receptor blockers (ARBs) for the treatment of hypertension and heart disease. The slow-offset ARBs candesartan and olmesartan show increased maximal efficacy compared to the fast-offset antagonist losartan, which has a 15–25-fold shorter ROt_1/2_ [37], [42]. It is thus not surprising that kinetic investigations are becoming a widespread practice in modern drug discovery and may either use biochemical assays to characterize compound-target complexes or – to increase functional relevance – employ functional assays [43].

In the present study, using second messenger assays in human primary PASMC, we characterized the inhibitory potency, kinetics and mode of antagonism of macitentan, which recently concluded testing in a Phase III, long-term, event-driven, morbidity/mortality clinical trial in patients suffering from PAH. PASMC are known target cells for successful PAH therapies and endothelin-mediated effects on PASMCs were shown to contribute to PAH pathology. Two ERAs, ambrisentan and bosentan, are currently approved PAH therapies and therefore the molecular receptor inhibition properties of macitentan were compared with those of ambrisentan and bosentan.

The ET-1-induced calcium signaling in human primary PASMC was largely ET_A_ driven, which was shown by using endothelin receptor subtype specific reference antagonists. The observed functional contribution of the ET_A_ receptor subtype in comparison to the ET_B_ receptor subtype was in agreement with our quantitative assessments of subtype specific mRNA expression and in agreement with the previously published reports on ET_A_/ET_B_ expression ratios in human PASMCs [28]. In this disease-relevant primary cell system macitentan, but not ambrisentan and bosentan displayed slow receptor dissociation causing insurmountable antagonism in ET-1-induced IP_1_ accumulation and ET-1-induced sustained elevation of intracellular calcium levels when ET-1 stimulation times were limited. Allowing for full equilibration of agonist and antagonist (90 min co-incubation) revealed the competitive nature of the antagonism exerted by macitentan. Our findings using second messenger assays in human primary PASMC confirm the competitive antagonism of macitentan previously shown in rat aortic ring contraction assays [30]. Since the development of vessel contraction by exogenously added ET-1 is a slow process (>100 minutes to reach EC_50_, data not shown), contraction assays with exogenously added ET-1 do not capture the insurmountability of macitentan which occurs with short ET-1 stimulation times only.

Macitentan and ambrisentan had the same antagonistic potency in steady state assays, but showed different kinetic properties. The likely reason for this difference is different ET_A_ binding modes of the molecules which are a function of their molecular structure. The structure of macitentan has nothing in common with the one of ambrisentan and differs substantially from the one of bosentan, and therefore different binding modes can be expected. The ET_A_ receptor has been suggested to have a bitopic ET-1 binding site for accommodation of the N-terminal ET-1 address-domain and the C-terminal receptor activation domain [44]. It has furthermore been suggested that low molecular weight ERAs might display allosteric antagonism or orthosteric antagonism. The data we obtained on macitentan, ambrisentan and bosentan are highly compatible with a direct competition of these ERAs with the binding of ET-1 to either of the two parts of the bitopic binding site. Due to the kinetic and structural differences we assume that the macitentan binding site is at least partially different from the bosentan and ambrisentan binding site and that this difference in binding contributes to the slow receptor dissociation of macitentan leading to insurmountable antagonism under hemi-equilibrium conditions. A combination of molecular modeling, site directed mutagenesis, NMR and X-ray studies would be required to prove this hypothesis.

### Direct Pathological Relevance in PAH of Sustained Elevation of Intracellular Calcium Levels in PASMC

This study differentiates macitentan and its mode of endothelin receptor antagonism from other ERAs using therapeutic target cells and measuring ET-1-induced second messenger levels, including the disease-relevant sustained elevation of cytosolic calcium concentrations. This sustained calcium elevation has been directly linked to sustained vessel contraction and pathological PASMC proliferation [29], [36]. In PASMC, the sustained increase in cytosolic calcium is mediated via a variety of plasma membrane channels including voltage-dependent channels, store-operated channels and transient receptor potential cation channels (TRPC). Interestingly, in proliferating PASMC or PASMC from idiopathic PAH patients higher intracellular calcium levels have been observed than in growth-arrested PASMCs or PASMCs from healthy subjects [45], [46], [47]. Specifically, TRPC channels have been shown to be upregulated in PAH-derived PASMC. Sustained calcium elevation is intimately linked to cellular proliferation by activation of mitogen activated protein kinases and inducing the pro-proliferative transcription factors c-jun and c-fos. Calcium also directly promotes cell cycle progression through formation of nuclear calcium-calmodulin complexes [48], [49]. Therefore, highly effective blockade of ET-1-induced sustained calcium release by a slowly dissociating ERA is expected to be beneficial in inhibiting pathological proliferation of PASMC.

### Advantages of a Slowly Dissociating Antagonist in the Blockade of the Paracrine/autocrine ET System

ET-1 is a paracrine/autocrine agonist released by endothelial cells, fibroblasts and smooth muscle cells in response to local stimuli such as hypoxia, growth factors or flow- and shear-stress and endothelial injury [20], [21], [22], [23], [24], [25]. Importantly, besides its constitutive secretion, ET-1 can also be released from specialized storage vesicles, so called Weibel-Palade bodies, which concentrate and store ET-1 beneath the plasma membrane of endothelial cells [17], [18]. Constitutive and stimulated ET-1 secretory mechanisms are complemented by highly effective local ET-1 degrading enzymes as evidenced by very short half lives of free ET-1 in tissue extracts [50], [51], [52], [53]. The spatial and temporal pattern of ET-1 release and degradation in diseased tissue and the resulting local ET-1 concentration-time profiles encountered by PASMC, cardiomyocytes, cardiac and lung fibroblasts are still unknown. However, under the assumption of fluctuating, i.e. not equilibrated local ET-1 concentrations in vivo, a slowly dissociating antagonist such as macitentan is expected to display a more complete block of ET-1 binding to its receptors than ambrisentan and bosentan. Furthermore, it is likely that the ET-1 that is prevented from binding to the blocked ET receptors is rapidly degraded and not available for renewed binding attempts.

### Conclusions

Macitentan is differentiated from the two currently approved ERAs through its slower receptor dissociation kinetics and insurmountable antagonism in non-equilibrium assays. Under conditions of local ET-1 fluctuations *in vivo*, macitentan should block ET-1-induced signaling more effectively than other ERAs. These qualities may contribute to a unique effectiveness of macitentan in diseases characterized by increased autocrine and paracrine ET-1 signaling such as PAH.

## Materials and Methods

### Cell Culture

Chinese hamster ovary (CHO) cells recombinantly expressing ET_A_ or ET_B_ receptors were cultivated in growth medium [Ham F-12 with L-glutamine containing 1000 µg/mL G418, 100 U/mL penicillin, 100 µg/mL streptomycin, and 10% heat-inactivated fetal calf serum (FCS)]. PASMC were purchased from TCS Cellworks (ZHC-3116) and cultivated in growth medium (Lonza Clonetics SmBM (CC-3181)) with SmGM-2 SingleQuots supplements and 5% FCS (CC-4149)). PASMC of passage 3 to 5 were used for all experiments.

### ERAs

Macitentan, bosentan, ambrisentan, and tezosentan were synthesized by Actelion Pharmaceuticals Ltd., sitaxentan was purchased from Convertex (Germany) and zibotentan from Acesys Pharmatech (USA). BQ-123 and BQ-788 were or purchased from Sigma.

### Intracellular Calcium Release Measurements Using Recombinant CHO-ET_A_ and CHO-ET_B_ Cells

CHO-ET_A_ or CHO-ET_B_ cells were seeded in growth medium at 20,000 cells/well into 384-well black clear-bottom sterile plates (Greiner) and incubated overnight at 37°C in 5% CO_2_. Then, the growth medium was exchanged by 50 µL/well of dye buffer [HBSS, 0.1% bovine serum albumin (BSA), 20 mM HEPES, 0.375 g/L NaHCO_3_, 5 mM probenecid (Sigma), 1% FCS and 3 µM fluo-4 AM]. Cells were incubated for 1 h at 37°C in 5% CO_2_ followed by equilibration at room temperature for at least 30 min. Then, within the Fluorescent Imaging Plate Reader (FLIPR Tetra, Molecular Devices), plates were subjected to protocols consisting of two additions of 10 µL each for the determination of the inhibitory potency (IC_50_ and K_b_) of the endothelin receptor antagonists. Calcium traces were recorded and then processed as described below. For the determination of antagonistic potency of endothelin receptor antagonists (K_b_) cells were supplemented with 10 µL of 6× concentrated antagonist dilution series prepared in assay buffer covering the final concentration range from 0 nM to 10 µM (0.5% DMSO final assay concentration). After a 120-min incubation at room temperature, cells were stimulated by the addition of 10 µL of 7× concentrated ET-1 to obtain an assay concentration of EC_50_–EC_70_. Calcium release was monitored for 3 min. For the K_b_ calculations, FLIPR traces were subjected to spatial uniformity correction and normalized by trace alignment at the last time point before agonist addition (ScreenWorks software, Molecular Devices). Then, relative fluorescence units (RFU) of the maximum signal per well were exported and used by the proprietary IC_50_ Witch software to calculate IC_50_ values (settings for IC_50_ values = use of fixed minimum (10 µM macitentan) and curve-intrinsic maximum). K_b_ values were calculated via the Cheng-Prusoff equation [K_b_ = IC_50_/(1+ [ET−1_stim_]/EC_50_ET−1_)] using the determined IC_50_ values, the EC_50_ of ET-1 (determined daily), and the ET-1 stimulating concentration used [31].

### Intracellular Calcium Release Measurements Using Human PASMC

PASMC were seeded in growth medium at 8,000 cells/well (exception sustained calcium release, see below) into 384-well black clear-bottom sterile plates (Greiner) and incubated overnight at 37°C in 5% CO_2_. Then, cell culture medium was exchanged by 50 µL/well of dye buffer [HBSS, 0.1% BSA, 20 mM HEPES, 0.375 g/L NaHCO_3_, 5 mM probenecid (Sigma) and 3 µM of fluo-4 AM]. The cell plates were incubated for 1 h at 37°C in 5% CO_2_ followed by equilibration at room temperature for at least 30 min. Within the FLIPR, plates were subjected to protocols consisting of two additions of 10 µL each, either for the determination of the inhibitory potency (IC_50_ and K_b_) of the endothelin receptor antagonists, for the analysis of antagonist ROt_1/2_, or for the analysis of sustained calcium fluxes. Calcium traces were recorded and processed as described below. For the K_b_ determinations, cells were supplemented with 10 µL of 6× concentrated antagonist dilution series prepared in assay buffer covering the final concentration range from 0 nM to 1 µM. The final assay concentration of DMSO was 0.1%. After 10 min or 120 min at room temperature, cells were stimulated with ET-1 by the addition of 10 µL of 7× concentrated ET-1 to obtain a final concentration of EC_50_–EC_70_. Calcium release was monitored for 3 min. For the K_b_ calculations, FLIPR traces were subjected to spatial uniformity correction and normalized by trace alignment at the last time point before agonist addition (ScreenWorks software, Molecular Devices). Then, RFU of the maximum signal per well were exported and used by the proprietary IC_50_ Witch software to calculate IC_50_ values [settings for IC_50_ values = use of fixed minimum (1 µM macitentan, always causing full signal blockade) and curve-intrinsic maximum]. K_b_ values were calculated via the Cheng-Prusoff equation using the determined IC_50_ values, the acutely determined EC_50_ of ET-1, and the ET-1 stimulating concentration used. For the determination of ROt_1/2_ values of the different ERA, cells were supplemented with 10 µL of antagonist dilution series and incubated at room temperature for 120 min. Then, cells were either stimulated in the FLIPR by the addition of 10 µL of 7× concentrated ET-1 (final assay concentration EC_50_–EC_70_), or cells were washed twice with 50 µL/well assay buffer (HBSS containing 0.1% BSA, 20 mM HEPES, 0.375 g/L NaHC0_3_, 2.5 mM probenecid, pH 7.4). After 5, 10, 20, 30 and 60 min of incubation at room temperature, cells were stimulated with an EC_50_–EC_70_ of ET-1 by the addition of 10 µL of a 7× concentrated ET-1 stock in assay buffer. Calcium release was monitored for 3 min. For the estimation of receptor ROt_1/2_, the K_b_ values were calculated via the Cheng-Prusoff equation using on-plate generated EC_50_ values of ET-1, and the earliest time point with a K_b_ value significantly shifted versus the non-washed control K_b_ value (K_b0__min_) was used. Significant change from K_b0__min_ (p<0.05) was determined using the one-way ANOVA test including Dunnett’s post test using GraphPadPrism software and is indicated with an asterisk in Fig. 3. An approximate ROt_1/2_ was then calculated assuming first order dissociation kinetics: t_1/2_ = t_x_/[log_2_ (K_bxmin_/K_b0__min_)] with x = time point of first significant change in K_b_ after wash-out.

For the determination of antagonistic effects on ET-1-induced sustained intracellular calcium release, PASMC were seeded in growth medium at 20,000 cells/well into FLIPR plates. After 1.5 h of attachment at 37°C/5% CO_2_, medium was exchanged with 50 µL/well of starvation medium (SmBM containing 1/10 of usual supplement concentrations). The next day, cells were dyed as described above and then supplemented with 10 µL of 6× concentrated dilution series of ERAs in assay buffer covering assay concentrations between 0 nM and 1 µM. The final DMSO concentration in the assay was 0.1%. After 120 min of incubation at room temperature, cells were stimulated by the addition of 10 µL of a 7× concentrated dilution series of ET-1 in assay buffer covering assay concentrations between 0 nM and 1 µM. Assay buffer in this assay type contained 2.5 mM probenecid. Intracellular calcium elevation was monitored for 25 min. The ERA effects on the first response phase were analyzed by calculating the K_b_ value from the IC_50_ generated at 8 nM ET-1 concentation. For the analysis of sustained calcium-response, FLIPR traces were subjected to spatial uniformity correction and normalized by trace alignment at the last time point before agonist addition (ScreenWorks software, Molecular Devices). Sustained calcium release was quantified by calculating the area under the FLIPR trace (area under the curve = AUC) between 3 min and 23 min after ET-1 addition. In addition, the IC_50_ value at 1.6 nM ET-1 stimulating concentration was determined using the proprietary IC_50_ Witch software (settings for IC_50_ values = use of fixed minimum (non-stimulated control) and curve-intrinsic maximum). K_b_ values were calculated via the Cheng-Prusoff equation using the determined IC_50_ values, the acutely determined EC_50_ of ET-1, and the ET-1 stimulating concentration used (1.6 nM).

### IP_1_ Measurements

All measurements were performed using the IP-One HTRF kit (Cisbio, #62IPAPEC) following the manufactureŕs protocol. PASMC were seeded at 10,000 cells/20 µL/well into 384-well, white, small-volume, tissue culture sterile plates (Greiner #784080) and incubated overnight at 37°C in 5% CO_2_. Then, medium was removed and 5 µL/well of assay buffer (HBSS containing 0.1% BSA, 20 mM HEPES, 0.375 g/L NaHCO_3_, 50 mM LiCl, pH 7.4) were added. Then, cells were supplemented with 5 µL/well of a 2× concentrated dilution series of antagonists in assay buffer covering a fincal concentration range of 0–10 µM and incubated for 120 min at 37°C in 5% CO_2_. Then, 2.5 µL/well of 5× concentrated solutions of ET-1 in assay buffer covering a final concentration range of 0–5 µM were added followed by an incubation at 37°C/5% CO_2_ for either 20 min or 90 min. Cells were lysed by the addition of 2.5 µL/well of conjugate-lysis buffer. Then, 2 µL/well of IP_1_-d2 conjugate and 3 µL/well of anti-IP_1_-cryptate terbium were added, and after 60 min incubation at room temperature, the assay plates were excited at 337 nm and the ratio of emitted light at 665 nm and 620 nm was recorded using the PHERAstar (BMG labtech). 665 nm/620 nm emission ratios were translated into IP_1_ concentrations via an on-plate calibration curve generated with known concentrations of IP_1._ IP_1_ concentration values were exported into GraphPadPrism software and the ET-1 concentration-response curves (CRC) in the presence of different antagonist concentrations were fitted by non-linear regression using the Gaddum/Schild analysis function for surmountable antagonism. The ET-1 CRC after 20-min stimulation in the presence of macitentan showed insurmountable antagonism and were fitted by the 3-parameter non-linear regression function of GraphPadPrism. To average the independent experiments, IP_1_ concentrations were normalized per experiment using the maximal response to ET-1 in the absence of antagonist as reference point. K_b_ values for the 20-min stimulation with ET-1 were obtained via the Cheng-Prusoff equation from the calculated IC_50_ value at 1.6 nM ET-1 [settings for IC_50_ calculation: curve intrinsic minimum (non-stimulated control) and curve-intrinsic maximum], the EC_50_ of ET-1 and the stimulating ET-1 concentration (1.6 nM). Schild K_b_ values for the 90-min stimulation with ET-1 (all ERAs were surmountable) were generated by the GraphPadPrism software after applying the Gaddum/Schild non-linear regression function. K_b_ values are expressed as geometric means of 3–4 independent measurements including the geometric standard deviation σ_g_ which is calculated from the standard deviation SD of the pK_b_ values as follows σ_g_ = 10exp(SD_pKb_).
